# Roles of Gut Microbiome in Bone Homeostasis and Its Relationship with Bone-Related Diseases

**DOI:** 10.3390/biology11101402

**Published:** 2022-09-26

**Authors:** Nina Zemanova, Radoslav Omelka, Vladimira Mondockova, Veronika Kovacova, Monika Martiniakova

**Affiliations:** 1 Department of Botany and Genetics, Faculty of Natural Sciences and Informatics, Constantine the Philosopher University in Nitra, 949 74 Nitra, Slovakia; 2 Department of Zoology and Anthropology, Faculty of Natural Sciences and Informatics, Constantine the Philosopher University in Nitra, 949 74 Nitra, Slovakia

**Keywords:** gut microbiome, bone homeostasis, osteoporosis, osteoarthritis, rheumatoid arthritis, diabetes mellitus, obesity, bone cancer, probiotic therapy, pharmacological drugs

## Abstract

**Simple Summary:**

In recent years, there has been increasing evidence that communication between the skeletal system and the gut microbiome (GM) can influence bone health and that the GM is a key regulator of bone homeostasis. Here, we review the roles of GM in bone homeostasis. In addition, the relationship between GM composition and selected bone-related diseases (osteoporosis, osteoarthritis, rheumatoid arthritis, diabetes mellitus, obesity and bone cancer) is presented. It is also emphasized that a probiotic supplementation can play an important role in suppressing the symptoms of each of these diseases.

**Abstract:**

The extended microbial genome—the gut microbiome (GM)—plays a significant role in host health and disease. It is able to influence a number of physiological functions. During dysbiosis, GM is associated with the development of various chronic diseases with impaired bone quality. In general, GM is important for bone homeostasis and can affect it via several mechanisms. This review describes the roles of GM in bone homeostasis through influencing the immune and endocrine functions, short-chain fatty acids production, calcium absorption and the gut–brain axis. The relationship between GM composition and several bone-related diseases, specifically osteoporosis, osteoarthritis, rheumatoid arthritis, diabetes mellitus, obesity and bone cancer, is also highlighted and summarized. GM manipulation may become a future adjuvant therapy in the prevention of many chronic diseases. Therefore, the beneficial effects of probiotic therapy to improve the health status of individuals with aforementioned diseases are provided, but further studies are needed to clearly confirm its effectiveness. Recent evidence suggests that GM is responsible for direct and indirect effects on drug efficacy. Accordingly, various GM alterations and interactions related to the treatment of bone-related diseases are mentioned as well.

## 1. Introduction

The gut microbiota is a unique and dynamic community of microorganisms residing in a specific environment—the gastrointestinal tract (GIT) [1]. The gut microbiome (GM), often used as a synonym for gut microbiota, is defined as a set of all genomic elements of a specific microbiota, representing an “extended genome” of millions of microbial genes. This microbial community consists not only of bacteria but also of archaea, viruses and unicellular eukaryotes [2].

The extended microbial genome—the microbiome—plays an important role in host health and disease [3]. The microbiome is able to affect numerous physiological functions, mostly through microbial metabolites, (e.g., nutrient and xenobiotic metabolism, protection against pathogens and regulation of the immune system and enteric nervous system) [4]. However, the gut microbiota is not stable over time, and factors that have been shown to alter the GM include aging, diet, environment or chronic treatment with oral antibiotics [5]. In general, decreased microbial diversity can be associated with dysbiosis (defined as an abnormal ratio of commensal and pathogenic bacteria), as there is a link between microbiome dysbiosis and various diseases [6,7].

As mentioned above, GM is able to produce multiple compounds that reach the circulation; hence, they can influence the functions of distal organs [8]. Nowadays, there is a growing interest in revealing the role of the microbiota in human health. Interestingly, there is a link between GM and skeletal health, and the microbiome also contributes to the regulation of bone homeostasis [9]. The GM–bone axis refers to the communication between microbial community and their metabolites on bone health. New findings support the information that microbiota is able to impact bone mineral density (BMD) and strength parameters. This can lead to a possibility to use beneficial bacteria as a future adjuvant probiotic therapy [10].

In this review, special emphasis is given to the relationship between GM composition and several chronic diseases consistent with impaired bone mass and bone quality, such as osteoporosis, osteoarthritis, rheumatoid arthritis, diabetes mellitus, obesity and bone cancer. Favorable impacts of probiotic therapy to alleviate symptoms in the above-mentioned disorders are also emphasized. The roles of GM in bone homeostasis through several mechanisms are reported as well. After all, various GM changes and interactions associated with the treatment of bone-related diseases are listed.

## 2. Materials and Methods

For this review, the PubMed database was used for the search of scientific articles. All searches were up to date as of 2022, and the search was conducted between March and August 2022. The search terms used included: “gut microbiome”, “bone-related diseases”, “bone health”, “bone diseases”, “bone homeostasis”, “osteoporosis”, “diabetes mellitus”, “osteoarthritis”, “rheumatoid arthritis”, “bone cancer”, “primary bone cancer”, “obesity”, “probiotics”, “probiotic supplementation”, “treatment” and “therapy”. Additionally, the combination of aforementioned keywords was used, and these combinations were connected with “and” (combinations of basic term “gut microbiome” and relevant disease, supplement or treatment) and “or” (between terms). The articles not related to the aims of the review were excluded. After analyzing the abstracts, the full text of each paper was checked. Only articles written in English were selected.

## 3. Composition and Functions of Gut Microbiome

GM is a dynamic system that is able to change over time. The first and most important step is a vertical transmission of maternal microbiota. This microbiota transmission and, subsequently, the colonization process of infants are influenced by the mode of delivery. Infants born by vaginal delivery, the most common mode of delivery, have colonization reflecting the maternal vaginal flora (*Lactobacillus* and *Prevotella* species). On the other hand, the microbiome of infants born by cesarean delivery consists of epidermal species (such as *Clostridium*, *Staphylococcus*, *Propionobacterium* and *Corynebacterium*) rather than vaginal ones, with lower numbers of *Bacteroides* and *Bifidobacterium* [11]. The GM undergoes rapid changes during infancy and early childhood and may also be formed by other factors, such as diet and antibiotics [12]. Another important factor influencing the GM of infants is the mode of feeding. The breastfed infant microbiome has a significantly higher numbers of *Bifidobacteria* and *Lactobacillus* and lower numbers of *Bacteroides*, *Staphylococcus* and *Enterobacteriaceae* in comparison with formula-fed infants. In addition, breast milk is a source of oligosaccharides that have a prebiotic effect [13]. Several studies [12,14,15] indicate that the GM continues to develop with a gradual increase in microbial diversity into adolescence. The pre-adolescent microbiome is enriched with *Bifidobacterium* spp., *Faecalibacterium* spp. and members of the *Lachnospiraceae* family [12]. In healthy adults, the microbiome is considered stable until old age (65 or older) and is shaped by environmental factors, such as diet and medication, rather than the genetics [12,16]. For example, animal-based diet is able to increase the abundance of bile-tolerant microorganisms (*Alistipes, Bilophila* and *Bacteroides*), reflecting an increased need of protein fermentation. On the other hand, in herbivorous mammals, there is a decreased level of *Firmicutes* (*Roseburia, Eubacterium rectale* and *Ruminococcus bromii*) metabolizing dietary plant polysaccharides [17]. In addition, diets high in fat, mainly in saturated fats, increase the abundance of Firmicutes, Proteobacteria and *Bilophila* spp. [18]. The microbial composition can also be affected by specific drugs, as several studies have reported [19]. Interestingly, changes in composition and diversity are associated with biological (functional) age, independent of chronological age (e.g., co-abundance module consisting of *Ruminococcus, Coprobacillus* and *Eggerthella* genera becomes abundant). However, with increasing biological age, the microbiota richness decreases [20].

In healthy individuals, most microbes inhabiting the GIT belong to two major phyla—Firmicutes and Bacteroidetes—representing more than 90% of relative abundance of the gut microbiota, followed by Actinobacteria, Proteobacteria, Synergistetes, Fusobacteria and others [21]. The Firmicutes phylum includes genera such as *Bacillus*, *Lactobacillus*, *Clostridium*, *Enterococcus* and *Ruminicoccus*. The Bacteroidetes phylum predominantly consists of *Bacteroides* and *Prevotella* [22]. However, even among the healthy individuals, the composition of the microbiota may vary, because each individual has their own unique microbiome. Hence, to define a healthy GM, we need to look at the functional level, as most people carry the same amount of bacterial genes involved in metabolic pathways [23].

**Figure 1:**
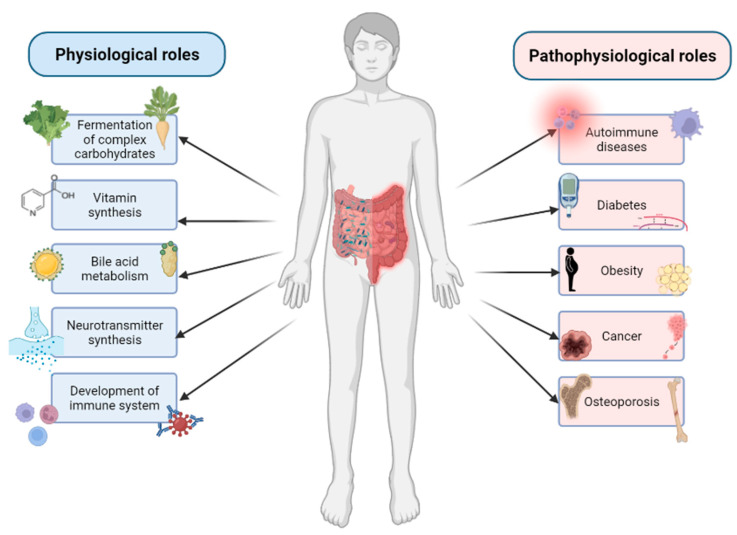
The GM has various roles in the host organism. It can affect various physiological roles, such as fermentation of complex carbohydrates [24], immune system [25], synthesis of neuroactive compounds [26], vitamin synthesis [27] and bile acid metabolism [28]. It can also affect many pathophysiological (during dysbiosis) roles [7]. (Created with BioRender.com).

The GM is able to affect the host organism and play a significant role in many physiological functions (Figure 1).

For example, specific dominant bacterial species are known for the fermentation of complex dietary carbohydrates, and some of more nutritionally specialized bacteria are also able to initiate degradation of plant cell walls, starch particles and mucin [24]. The gut microbiota can also affect the largest immunological organ—the GIT. GIT is not only the main line of defense against pathogens, limiting their direct contact with the epithelium, but the bacterial community of GIT is also necessary for the development of a fully functional immune system [25]. In addition, the intestinal microbiota is capable to affect the central nervous system through multiple bidirectional pathways involving neural, endocrine and immune signaling and is known as gut–brain axis. The microbiota can produce neurotransmitters and secrete neuroactive metabolites [26]. It can also synthesize certain vitamins, namely vitamin K and B group vitamins, such as thiamine (B_1_), riboflavin (B_2_), niacin (B_3_), pantothenic acid (B_5_), pyridoxine (B_6_), biotin (B_7_), folate (B_9_) and cobalamin (B_12_). The synthesis of these vitamins is not only important for intestinal bacteria, but there is also a metabolic and physiological significance of these pathways in mammals [27]. The GM also plays an important role in the bile acid metabolism. Gut bacteria contribute to the salvage of bile salts that escape active transport in the distal ileum. In addition, secondary bile acids (deoxycholic acid and lithocholic acid) are produced solely by bacteria in the human large intestine [28]. However, during dysbiosis, the GM is associated with development of various metabolic and immune diseases, including obesity, diabetes, inflammatory bowel disease, Crohn’s disease, cancer, allergies and osteoporosis (Figure 1) [7].

## 4. The Role of Gut Microbiome in Bone Homeostasis

The skeleton forms a structural support of vertebrates with the ability to resist the mechanical forces. It has mechanisms to grow and change its shape and size, as bone tissue is continuously formed and remodeled via two separate processes—bone resorption and bone formation—managed by osteoclasts (OCs) and osteoblasts (Obs) [29,30]. Bone homeostasis is a dynamic process, regulated by complex molecular pathways, where Ocs resorb bone tissue and Obs produce new bone tissue; thus, the net bone mass is maintained. Additionally, bone marrow endothelial cells were found to have a role in regulating bone homeostasis [31]. The bone is also a major source of inorganic ions and actively participates in calcium and phosphate balance [30]. In addition, the bone is a metabolically active organ and is important for the immune system, as immune cells originate from bone marrow, where many of them also mature [32]. Generally, GM can affect bone homeostasis by influencing immune function, host metabolism, hormone secretion and the gut–brain axis.

### 4.1. GM Influencing Bone Homeostasis via Immune Function

**Figure 2:**
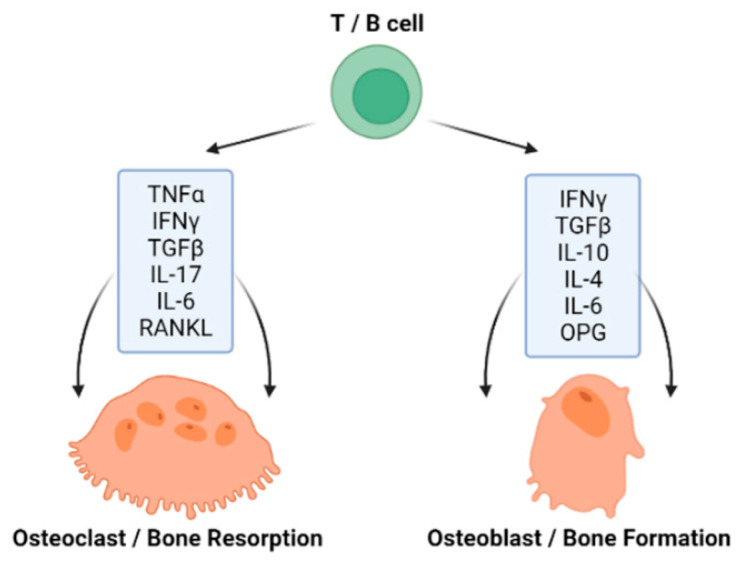
Lymphocytes can regulate bone remodeling through the expression of cytokines, as well as RANKL and OPG. (Created with BioRender.com).

The immune system represents a complex network of cells, compounds and processes that defend and protect the organism from foreign antigens and is tightly connected with bone homeostasis (Figure 2) [33]. An acquired immunity and the skeletal system have been recorded to evolve simultaneously, as there are molecules, such as receptor activator of nuclear factor κ-B ligand (RANKL) and the tumor necrosis factor (TNF) family, which have a significant role in both systems [34]. T cells (T-lymphocytes) are involved in both bone resorption and bone formation through the expression of pro- and anti-inflammatory cytokines, as well as RANKL and OPG [35]. Interestingly, B cells (B-lymphocytes) also produce the key osteoclastogenic cytokine RANKL and its physiological inhibitor osteoprotegerin (OPG) [36,37]. The process of OCs formation requires specific cytokines, primarily RANKL; macrophage colony-stimulating factor (M-CSF); TNF-α; interleukin (IL)-1, IL-7, IL-17, IL-23 and IL-6; interferon (IFN) γ and transforming growth factor-β (TGF-β). OBs are able to express the parathyroid hormone (PTH) receptor, which, upon ligand binding, can activate OC activity. OBs also produce RANKL and OPG. While RANKL stimulates osteoclastogenesis, OPG inhibits this effect [38]. The process of OB formation also requires specific T-lymphocyte cytokines such as IL-10, IL-4, IL-6 and IFN γ [37]. 

The GM has a great impact on the host organism, so it is not surprising that it also contributes to the regulation of bone homeostasis. This homeostasis is also affected by the immune system; hence, there is a close link between immunity and gut microbiota [9]. The relationship between gut microbiota, immune system and bone health is a relatively new area of study, as Ohlsson and Sjögren [39] even proposed a new interdisciplinary research field called “osteomicrobiology”, combining bone physiology, gastroenterology, immunology and microbiology.

**Figure 3:**
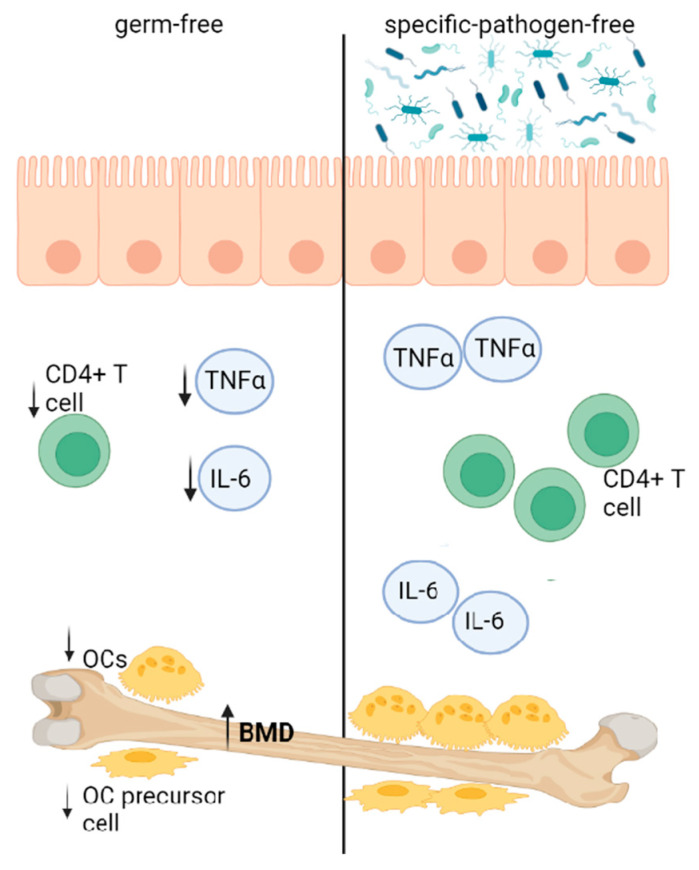
Mice lacking GM exhibited increased trabecular BMD, which was associated with decreased OCs, CD4^+^ T cells and OC precursor cells and lowered TNFα and IL-6 expression [40]. T cells are known to secrete osteoclastogenic cytokines such as TNF- α and IL-6 [41]. Intestinal bacteria are needed to develop the immune system. In GF mice, the mucosal immune system is undeveloped, having reduced the lamina propria CD4^+^ T cells. Moreover, the spleen and lymph nodes are also affected, as they are relatively structureless with poorly formed B- and T-cell zones [42]. (Created with BioRender.com).

In general, the gut microbiota can affect bone quality and quantity. In the study by Sjögren et al. [40], germ-free (GF) mice exhibited increased trabecular BMD. A higher bone mass was associated with decreased numbers of OCs, CD4^+^ T cells and OC precursor cells and lowered TNFα and IL-6 expression. Interestingly, the colonization of GF mice resulted in normal bone mass levels (Figure 3).

Another study looked at the possibility of innate immune signaling pathways mediating the effect of the microbiota. This was investigated in GF and CONV-R (conventionally raised) mice with the targeted inactivation of MYD88, NOD1 or NOD2 [43]. NOD1 and NOD-2 are specialized NOD-like receptors, and they have an important role in recognizing pathogens and activating immune responses [44]. On the other hand, myeloid differentiation factor 88 (MyD88) is used by toll-like receptors (TLRs) and activates NF-kB and mitogen-activated protein kinases (MAPKs) to induce the production of inflammatory cytokines [45]. The study has shown that the cortical bone mass was elevated in wild-type and MyD88-deficient GF mice compared to the corresponding CONV-R mice. This increase was not detected in the Nod1^−/−^ or Nod2^−/−^ GF mice. The bone expression of TNFα and RANKL was reduced in GF mice (compared to CONV-R wild-type mice). However, this trend was not observed in Nod1^−/−^ or Nod2^−/−^ mice. This suggests that the effect of gut microbiota on the bone mass depends on the NOD1 and NOD2 signaling pathways [43]. On the contrary, Schwarzer and colleagues [46] demonstrated that wild-type microbiota is associated with optimal systemic somatic growth, as the microbiota influences skeletal growth. The bone growth parameters were decreased in GF animals, although the cortical BMD was not affected. In addition, insulin-like growth factor 1 (IGF-1) was significantly reduced. These contradictory findings may be a result of several differences between animals (different sex, age or strain) in published research.

### 4.2. GM Influencing Bone Homeostasis via Short-Chain Fatty Acids Production

Microbial products such as bacterial fermentation molecules—short-chain fatty acids (SCFAs)—can also affect bone homeostasis. SCFAs (mainly acetate, propionate and butyrate) are products of carbohydrate fermentation that escape digestion and absorption in the small intestine [47]. 

Wallimann and colleagues [48] showed that butyrate can significantly reduce OC formation and resorption activity. They also demonstrated that butyrate supplementation can reduce systemic IL-6 levels in a murine model of osteotomy. In another study, Lucas et al. [49] also wanted to determine whether SCFAs are able to affect bone metabolism in two murine models of inflammatory arthritis. Butyrate and propionate have been shown to have a beneficial impact on bone homeostasis. The effect of these SCFAs depends on metabolic reprogramming of pre-osteoclasts, resulting in enhanced glycolysis at the expense of oxidative phosphorylation, leading to the downregulation of essential osteoclast genes such as TRAF6 and NFATc1. SCFAs can also help to alleviate inflammation by inducing T_reg_ cells.

### 4.3. GM Influencing Bone Homeostasis via Absorption of Calcium

GM can affect the absorption of nutrients related to skeletal development, such as calcium and vitamin D. The skeleton contains 99% of all calcium in the body [29]. The integrity of calcium and phosphate homeostatic mechanisms mediated by parathyroid hormone, fibroblast growth factor 23 and vitamin D is required for normal bone formation, metabolism and repair [50]. In the distal intestine (mostly the ileum), approximately 70–80% of the ingested calcium is absorbed and is facilitated by vitamin D—specifically, by 1, 25-dihydroxyvitamin D_3_. Calcium absorption involves a calcium influx, translocation of calcium through the enterocyte and basolateral extrusion of calcium by the intestinal plasma membrane pump [51].

A couple decades ago, it has been shown that the presence of SCFAs affected the epithelial tissue and promoted the calcium absorption from the large rat intestine in vitro (using an Ussing chamber) [52]. However, this study did not support a popular theory that a reduced pH environment (caused by the fermentation of prebiotic fiber to SCFAs) can help to elevate the absorption of calcium, because treating the rat colonic segments with HCl did not increase the calcium transport. Several studies have been focused on the effects of prebiotic supplementation (prebiotic dietary fibers, which are fermented by bacteria to SCFAs) and its interactions with mineral absorption. Bryk et al. [53] analyzed the effect of supplementing diets with the GOS/FOS^®^ mixture (a prebiotic mixture of 90% of short-chain galacto-oligosaccharides (GOS) and 10% long-chain fructo-oligosaccharides (FOS)) in growing rats. This mixture increased the bone mineralization and BMD due to higher calcium, phosphorus and magnesium absorptions. Another study investigated the impact of dietary inulin in gluten-free diet and its ability to influence the absorption of calcium [54]. Interestingly, the effect of inulin on mineral utilization depended on the dietary calcium intake from diet. Increased relative calcium absorption from inulin consumption was observed in rats fed with a calcium-restricted gluten-free diet. In addition, some studies also suggest the beneficial effects of prebiotics on calcium absorption in humans. For example, Whisner and colleagues [55] showed that soluble corn fiber (a nondigestible carbohydrate) can increase calcium absorption efficiency in adolescent males and females. A positive impact of a product rich in transgalacto-oligosaccharides (TOS) consumption on calcium absorption was also recorded in postmenopausal women [56]. The increase of calcium absorption was not accompanied by increased urinary excretion, meaning that TOS may indirectly elevate the uptake of calcium by bones and/or inhibit bone resorption.

### 4.4. GM Influencing Bone Homeostasis via Endocrine Function

The endocrine system (including parathyroid hormone, PTH-related peptide, calcitonin, vitamins A and D, estrogens, androgens and growth hormone) controls a balance between bone formation and bone loss and the maintenance of calcium and phosphate homeostasis [30]. In addition, there is evidence that osteocalcin is also required to stimulate bone mineral maturation [30].

Estrogen receptors are expressed in various tissues, including the brain, bone and adipose tissue; hence, estrogen is able to affect a variety of physiological responses, such as BMD [57]. Li et al. [58] showed that GM modulates the inflammatory responses (upregulated osteoclastogenic cytokines, such as TNFα, RANKL and IL-17) caused by sex steroid deficiency, which leads to trabecular bone loss in a murine model. On the other hand, in GF mice, sex steroid deficiency did not increase osteoclastogenic cytokines production. Additionally, an increase of epithelial barrier permeability and decreased expression of gut epithelial tight junction proteins were recorded.

Vitamin D is mainly known for its role in bone homeostasis (calcium and phosphate absorption), but it has various direct and indirect regulatory effects on the immune system (promoting T_reg_ cells, inhibiting differentiation of Th1 and Th17 cells, impairing the development and function of B cells and reducing monocyte activation) [59]. It has been shown that the GM can be altered by vitamin D. According to Ooi and colleagues [60], the absence of a vitamin D receptor and the ability to produce an active form of vitamin D are associated with inflammation of the intestine, which is caused by the expansion of *Proteobacteria* phylum (including the Helicobacteraceae family members). Hence, vitamin D supplementation could potentially protect against gastrointestinal injury. Another study, conducted by Luthold et al. [61], supported the effect of vitamin D on the commensal bacterial composition. Interestingly, vitamin D is also able to directly inhibit mycobacterial growth in culture [62]. 

Osteocalcin is a non-collagenous protein secreted by OBs containing three glutamate residues that can be carboxylated. This modification is catalyzed by γ-glutamyl carboxylase, depending on vitamin K, O_2_ and CO_2_ as the cofactors, supplied by the vitamin K cycle and circulation [63]. Carboxylated osteocalcin is essential for the alignment of apatite crystals and optimal bone strength [64]. As the GM has been suggested to contribute to the maintenance of vitamin K, Wagatsuma et al. [65] aimed to clarify the association between GM and alternative indicator of vitamin K deficiency (serum undercarboxylated osteocalcin concentration) in patients with Crohn’s disease. They recorded a significant negative correlation between serum undercarboxylated osteocalcin and mean Chao1 index (determining the alpha diversity).

### 4.5. GM Influencing Bone Homeostasis via the Gut–Brain Axis

The GM, namely certain bacteria (*Streptococcus*, *Corynebacterium* and *E. coli*), can produce various molecules, including monoamine neurotransmitters such as serotonin, which is a key neurotransmitter within both the enteric and central nervous systems [8]. 

Peripheral serotonin, produced primarily by the intestinal flora, functions as a hormone inhibiting OB proliferation through 5-hydroxytryptamine receptor 1B and the cAMP response element-binding protein, causing the decrease of BMD and bone formation [66]. On the other hand, brain-derived serotonin appears to be a positive regulator of bone development, acting via sympathetic tone, as well as a molecule regulating food intake and energy expenditure [67]. Supporting these findings, Sjögren et al. [40] showed that GF mice compared to CONV-R mice had lower levels of serum serotonin and an increased relative volume of trabecular bone. However, the colonization of GF mice led to a normalization of trabecular bone mass, but it has no significant effect on the serum serotonin levels.

In addition, tryptophan is a precursor to serotonin, and changes in the tryptophan levels were recorded in bone metabolic diseases [68]. The discovery of the exact role of tryptophan or tryptophan metabolites in bone may result in finding a new effective therapy for management of bone-related diseases. For example, D-tryptophan is a newly identified product from probiotic bacteria [69]; however, more studies are needed to clarify its effectiveness as prebiotic supplement.

## 5. The Relationship between Gut Microbiome and Bone-Related Diseases

Various diseases are accompanied by changes in GM composition [6]. Bone-related diseases can also be linked to altered microbiome, with significant changes in levels of phyla *Firmicutes*, *Bacteroidetes* and *Actinobacteria* [35]. Additionally, recent studies are focusing on the potential positive effects of GM modulators, such as probiotic and prebiotic supplementations, in both human and animal models. The following text describes in more detail the association between GM and several bone-related diseases, including osteoporosis, osteoarthritis and rheumatoid arthritis, diabetes mellitus (DM), obesity and bone cancer.

### 5.1. Osteoporosis

Osteoporosis is the most common skeletal disorder, affecting approximately 200 million individuals worldwide [50]. It is characterized by reduced BDM, the microstructural degradation of bone tissue and increased risk of fractures [69]. At the microstructural level, increased bone resorption and decreased bone formation simultaneously occur, leading to a decline in bone mass. The treatment of osteoporosis can be divided into four categories: lifestyle changes (e.g., dietary adjustment, exercise, smoking cessation and avoiding heavy alcohol consumption); nutritional supplements (increased calcium and vitamin D intake); pharmaceutical intervention-antiresorptive agents (e.g., bisphosphonates, selective estrogen receptor modulators, calcitonin and hormone therapy) and anabolic agents (such as parathyroid hormone analogues and strontium ranelate) and surgical management [70,71,72]. In addition, some studies support the idea of importance of circadian rhythmicity for bone health, as sleep disruption and circadian misalignment have been associated with lower BMD and increased fracture risk [73]. Although melatonin is mostly known as an important regulatory factor of circadian rhythm, there is a possibility of its function as a protector in bone injury and osteoporosis; therefore, melatonin supplementation might positively affect bone tissue [74]. Melatonin is secreted from the pineal gland; however, it is also synthesized in the intestines by enterochromaffin cells and gut microbes, where it acts locally rather than entering systemic circulation. There is more melatonin in the gut than in the pineal glands, which proves its importance to gut function [75]. Since melatonin is synthesized from tryptophan with the intermediate serotonin, some drugs such as selective serotonin reuptake inhibitors (SSRIs; the most commonly prescribed antidepressants) can interfere with melatonin production.

Several studies have evaluated the abundance and diversity of bacterial populations in the intestine of patients with osteoporosis (Table 1). Based on these, adults with osteoporosis appear to have a reduced diversity of microorganisms, with an increase in certain species such as *Fusobacterium, Dialister, Faecalibacterium* and *Tolumonas* and a decrease in *Bacteroides* and *Roseburia* spp. [76,77,78,79]. The GM may influence the risk of osteoporosis through effects on endogenous estrogens. The ratio of estrogen metabolites to parent estrogen was associated with relative abundances of a number of taxa in the class Clostridia (including the order Clostridiales and the family Ruminococcaceae), whereas the genus Bacteroides was inversely associated with this ratio [80]. Another study found [81] that a number of unique bacterial species is directly associated with systemic estrogens; thus, the GM could affect the risk for estrogen-related conditions in older adults.

The gut microbiota is able to affect bone health by modulating inflammatory actions caused by a lack of sex steroids. This deficiency can be observed in postmenopausal osteoporosis, where is mainly caused by ovarian function cessation, while a decline in estrogen levels results in increased bone resorption. In general, the GM regulates estrogens through secretion of β-glucuronidase, which deconjugates estrogens into their active forms. When this process is disrupted by dysbiosis, the decrease in deconjugation results in a reduction of circulating estrogens [57]. In ovariectomized (OVX) mice, elevated gut permeability; expanded Th17 cells and upregulated levels of osteoclastogenic cytokines (such as TNFα, RANKL and IL-17) were reported. On the contrary, sex steroid deficiencies did not affect the osteoclastogenic cytokines in GF mice [58]. 

Interestingly, there is also a close relationship between GM and vitamin D_3_. A recent study [82] has shown that vitamin D_3_ supplementation correlated with the alteration of gut microbiota towards a decrease in Firmicutes to Bacteroidetes ratio in adults with vitamin D insufficiency/deficiency.

Recently, there has been a great interest in probiotic supplementation and its positive effects on health. In general, probiotics are living microorganisms (species such as Lactobacilli, Bifidobacteria, Enterococcus and *Bacillus subtilis*) that are intended to have health benefits to the host [83]. Li and colleagues [84] focused on the effect of probiotic supplementation on bone loss in OVX mice. The treatment of OVX mice with *Lactobacillus rhamnosus* GG or commercially available probiotic supplement VSL#3 (containing eight strains of bacteria—*Bifidobacterium breve*, *B. longum*, *B. infantis*, *Lactobacillus acidophilus*, *L. plantarum*, *L. paracasei*, *L. bulgaricus* and *Streptococcus thermophilus*) reduced the gut permeability, intestinal and bone marrow inflammation and protected against bone loss [84]. Another study focused on the impact of probiotic treatment, using a mix of three *Lactobacillus* strains (*L. paracasei* DSM 13434, *L. plantarum* DSM 15312 and *L. plantarum* DSM 15313) in early postmenopausal women [85]. In general, *Lactobacillus* treatment reduced the lumbar spine BMD (LS-BMD) loss compared to the placebo group. Britton et al. [86] revealed a beneficial effect of *Lactobacillus reuteri ATCC PTA 6475* treatment in OVX mice. In these mice, a significant decrease of osteoclastogenesis (via the suppression of bone marrow CD4^+^ T lymphocytes), as well as bone resorption markers and activators (tartrate resistant acid phosphatase 5 (TRAcP5) and RANKL), were recorded. Moreover, there is also a study investigating the effect of the probiotic *Bacillus subtilis* C-3102 on BMD in healthy postmenopausal women [77]. The data suggest that 12 weeks of the probiotic supplementation resulted in a significant increase in relative abundance of the genus *Bifidobacterium* and in a significant decrease in relative abundance of the genus *Fusobacterium*. Additionally, there was an increase in total hip BMD in the *L. reuteri*-treated group, which correlates with a decrease of bone resorption markers (urinary type I collagen crosslinked N-telopeptide and TRAcP5b). 

**Table 1:** Summary table of various GM alterations associated with bone-related diseases and the effects of probiotic therapy used in animal and human studies.

Disease	GM Alterations Associated with the Disease	Probiotic Therapy Used	Effects of Probiotic Therapy in Studies	References
osteoporosis	HS: increase in *Fusobacterium*, *Dialister*, *Faecalibacterium*, *Tolumonas, Bacteroides, Parabacteroides, Adlercreutzia, Lactobacillus;* decrease in *Roseburia*, *Clostridia*, *Methanobrevibacter, Romboutsia, Turicibacter, Lachnospira*	AS: *Lactobacillus rhamnosus, L. acidophilus*, *L. plantarum*, *L. paracasei*, *L. bulgaricus*, *L. reuteri, Bifidobacterium breve*, *B. longum*, *B. infantis*, *Streptococcus thermophilus* HS: *Lactobacillus paracasei*, *L. plantarum*, *Bacillus subtilis*	AS: reduced gut permeability, intestinal and bone marrow inflammation; decrease of osteoclastogenesis and bone resorption markers HS: reduced lumbar spine BMD loss; increased total hip BMD; increased *Bifidobacterium*	[76,87,88,89]
osteoarthritis	HS: increase in *Clostidium, Gemmiger, Klebsiella, Akkermansia, Lactobacillus, Betaproteobacteria, Streptococcus, Bilophila, Desulfovibrio* decrease in *Bifidobacterium, Faecalibacterium, Bacteroides, Prevotella, Alistipes, Clostridium, Parabacteroides, Roseburia*	AS: *Clostridium butyricum, Lactobacillus acidophilus*, *L. casei*, *L. fermentum*, *L. paracasei*, *Streptococcus thermophilus*, *Bifidobacterium longum*, *B. bifidum, B. breve*, *L. rhamnosus*, *L. plantarum*, *L. helveticus*, *L. salivarius*	AS: preserved knee cartilage and synovial membrane; reduced fibrous tissue; decreased cartilage damage; lowered inflammatory and bone metabolism markers in serum; increased levels of IFN-γ and glycosaminoglycans; alleviated pain; increased *Bifidobacterium and Roseburia;* decreased *Closteridium leptum, Akkemansia muciniphila*	[90,91,92,93,94,95,96,97,98,99,100]
rheumatoid arthritis	HS: increase in *Prevotella, Clostridium, Ruminococcus*, *Lactobacillus, Collinsella, Eggerthella* decrease in *Bacteroides, Haemophillus, Firmicutes, Faecalibacterium,*	AS: *Lactobacillus casei* HS: *L. acidophilus*, *L. casei*, *B. bifidum*	AS: inhibited joint swelling, lowered arthritis scores, prevented bone destruction; downregulated pro-inflammatory cytokines; increased *L. acidophilus* HS: improved Disease Activity Score, decreased serum insulin	[101,102,103,104,105,106]
type 1 diabetes mellitus	HS: increase in *Bacteroides*, *Veillonella*, *Alistipes*, *Klebsiella*, *Enterococcus*, *Clostridium*, *Staphylococcus, Streptococcus, Sarcina, Corynebacterium, Barnesiella, Haemophilus,* *Ruminococcus*, *Blautia* decrease in *Bifidobacterium*, *Lactobacillus*, *Escherichia, Prevotella, Akkermansia, Eubacterium,* *Roseburia*, *Faecalibacterium*, *Lachnospira*	AS: *Lactobacillus brevis, L. reuteri, L. lactis, L. kefiranofaciens, L. kefiri, Bifidobacterium, Streptococcus thermophilus* HS: *L. paracasei, L. plantarum, L. acidophilus, L. delbrueckii, B. longum, B. infantis, B. breve, Streptococcus thermophiles*	AS: reduced blood glucose levels via gamma-aminobutyric acid; elevated innate response; reduced intestinal inflammation; suppressed osteoblast Wnt pathway; stimulated secretion of anti-inflammatory cytokines HS: decrease in glycated hemoglobin, decline in total and bolus insulin; ameliorated conditions of metabolic syndrome	[107,108,109,110,111,112]
type 2 diabetes mellitus	HS: increase in *Ruminococcus*, *Fusobacterium*, *Blautia, Bacteroides, Clostridium, Eggerthella, Escherichia* decrease in *Bifidobacterium*, *Bacteroides*, *Faecalibacterium*, *Akkermansia, Roseburia, Firmicutes*	AS: *Lactobacillus rhamnosus, L. casei, L. plantarum, L. acidophilus, L. paracasei, Bifidobacterium bifidum* HS: *L. casei, L. reuteri, L. acidophilus, Bifidobacterium lactis*	AS: decreased fasting and postprandial blood glucose; improved insulin sensitivity and reduced lipid accumulation by stimulating adiponectin secretion; reduced plasma lipids and pro-inflammatory cytokines HS: decreased fasting and postprandial blood glucose; reduced plasma lipids and pro-inflammatory cytokines	[109,113,114,115,116,117,118,119,120,121,122,123,124,125,126,127,128,129]
obesity	HS: increase in *Firmicutes, Acidaminococcus, Anaerococcus, Catenibacterium, Dialister, Dorea, Escherichia-Shigella, Eubacterium, Fusobacterium, Megasphera, Prevotella, Roseburia, Streptococcus, Sutterella, Staphylococcus, Clostridium, Lactobacillus* decrease in *Bacteroidetes, Bifidobacterium, Eggerthella*	AS: *Lactobacillus gasseri, L. plantarum, L. curvatus, L. reuteri, L. acidophilus*, *L. paracasei*, *L. bulgaricus*, *Bifidobacterium breve, B. pseudocatenulatum, B. longum, B. infantis*, *Streptococcus thermophiles* *HS:* *L. acidophilus, L. rhamnosus, L. gasseri, L. salivarius, L. casei, L. amylovorus L. fermentum, L. plantarum, Enterococcus faecium, Streptococcus thermophilus, Bifidobacterium lactis*	AS: decreased weight, body fat and leptin; decreased insulin resistance, triglyceridemia, cholesterolemia; increased trabecular bone volume and bone mechanical strength; improved osteoblast mineralization HS: decreased LDL cholesterol, body weight, BMI, visceral and subcutaneous fat, waist and hip circumference; increased plasma adiponectin	[130,131,132,133,134,135,136,137]

GM—gut microbiome; AS—animal studies; HS—human studies; BMD—bone mineral density; IFN—γ–interferon γ; LDL—low-density lipoprotein; BMI—body mass index.

### 5.2. Inflammatory Bone-Related Diseases

Inflammatory diseases involving bones and joints are very common in the world, as 14 million people worldwide suffer from rheumatoid arthritis. Primarily, joint diseases are characterized by a systemic osteoporosis and increased fracture rates [138]. Generally, these disorders are linked by the presence of an inflammatory process targeting the joints with adverse effects on the structure and function [139]. From this group, osteoarthritis and rheumatoid arthritis are described in more detail.

#### 5.2.1. Osteoarthritis

Osteoarthritis (OA) can be defined by joint symptoms, structural pathology or both [140]. It is characterized by degeneration of articular cartilage, leading to joint pain and disability [141]. OA is a multifactorial disorder; thus, both the systemic and local factors (e.g., age, sex, ethnic characteristics, BMD, genetics, obesity, joint injury and muscle weakness) must be taken into account [142]. The treatment of OA falls into four categories—nonpharmacological (exercise and modification of lifestyle); pharmacological (e.g., acetaminophen, non-steroidal anti-inflammatory drugs and intra-articular injections of corticosteroids or hyaluronic acid); complementary and alternative (e.g., glucosamine and chondroitin supplements and balneotherapy) and surgical ones. The treatment should begin with the least invasive therapy, and all patients should receive their treatment from the first two categories [99].

Several studies examined the differences in GM compositions between patients with OA and healthy individuals (Table 1). Chen et al. [93] revealed a decrease of *Bifidobacterium longum* and *Faecalibacterium prausnitzii* and an increase of *Clostidium* spp. in OA patients. This study correlated with the results of Bonato et al. [94], who noted that the *Clostridium* genus was upregulated in various studies. Interestingly, Wang and colleagues [95] analyzed stool samples from overweight OA patients and overweight healthy individuals by 16S rRNA gene sequencing. Their findings showed that the relative abundance of nine genera differed between the groups, as the genera of *Gemmiger, Klebsiella, Akkermansia* and *Lactobacillus* were enriched in OA patients, while *Bacteroides, Prevotella, Alistipes, Clostridium XI* and *Parabacteroides* were enriched in the control group.

There are numerous researchers suggesting the positive effects of probiotic supplementation in rats with OA. Sim et al. [96] found that *Clostridium butyricum* therapy prevented OA symptoms, preserved knee cartilage and synovial membrane and significantly decreased the amount of fibrous tissue. This supplementation also significantly lowered the serum levels of inflammatory and bone metabolism markers (cyclooxygenase-2, leukotriene B4, cartilage oligomeric matrix protein and IL-6) and increased the levels of IFN-γ and glycosaminoglycans. Another study [97] revealed that the combination with a probiotic complex (consisting of *Lactobacillus acidophilus*, *L. casei*, *L. fermentum*, *L. paracasei*, *Streptococcus thermophilus*, *Bifidobacterium longum*, *B. bifidum, B. breve*, *L. rhamnosus*, *L. plantarum*, *L. helveticus* and *L. salivarius*); rosavin (natural product found in *Cinnamomum iners*, *Cinnamomum aromaticum* and others) and zinc ameliorated pain by preventing cartilage damage in rats with OA. Additionally, the expression of proinflammatory cytokines was decreased. Lee and colleagues [98] showed that *Lactobacillus acidophilus* supplementation alleviated OA-associated pain and delayed the progression of this disease by inhibiting the levels of proinflammatory cytokines in the joints. Interestingly, the oral administration of *L. casei* together with type II collagen and glucosamine more effectively reduced pain, cartilage destruction and lymphocyte infiltration than the individual treatment of glucosamine or *L. casei*. Additionally, the expression of pro-inflammatory cytokines (such as IL-1β, IL-2, IL-6, IL-12, IL17, TNF-α and IFN-γ) and matrix metalloproteinases was decreased, while anti-inflammatory cytokines (IL-4 and IL-10) were increased [100]. According to a preliminary clinical study [143], *Streptococcus thermophilus* (TCI633), a bacterium able to produce hyaluronate in GIT, could improve the degeneration of knee osteoarthritis in humans.

#### 5.2.2. Rheumatoid Arthritis

Rheumatoid arthritis (RA) is a systemic inflammatory autoimmune disease characterized by synovial inflammation and hyperplasia; auto-antibody production and systemic features (cardiovascular, pulmonary and psychological disorders) [144]. It is also responsible for joints destructions associated with bone complications that include periarticular bone loss, bone erosions and systemic osteoporosis [139]. The most important factors in the management of RA are the early diagnosis, prompt disease-modifying anti-rheumatic drugs (DMARDs) therapy and monitoring of patients to increase the likelihood of remission. The current available drug therapy includes non-steroidal anti-inflammatory drugs (NSAIDs), glucocorticoids and DMARDs (such as methotrexate, Janus kinase inhibitors, TNF inhibitors, IL-6 inhibitors and B-cell depleting drugs). Non-pharmacological treatments such as physical therapy; lifestyle changes (e.g., smoking cessation, attaining ideal body weight and exercise) and surgical management belong to important treatment resources as well [102].

Scher et al. [103] identified the increase in *Prevotella copri* accompanied by a reduction in *Bacteroides* and a loss of beneficial microbes in untreated patients with RA. Zhang et al. [104] detected dysbiosis in the gut in these patients; however, it was partially resolved after the treatment. In the oral samples, there was an increase in *Porphyromonas gingivalis*, while the gut microbiota had elevated numbers of *Clostridium* spp., *Ruminococcus* spp. and *Lactobacillus* spp. and lower numbers of *Haemophillus* and *Firmicutes*. The information is presented in Table 1.

There is increasing evidence that probiotic supplementation could reverse a microbial disorder in patients with RA. The *L. casei* (ATCC334) treatment of adjuvant-induced arthritis (AIA) in rats inhibited joint swelling, lowered the arthritis scores and prevented bone destruction, while the relative abundance of *Lactobacillus* strains, which is decreased in AIA rats, was restored to normal, and the level of *L. acidophilus* was even increased. In addition, *L. casei* supplementation caused a downregulation of proinflammatory cytokines [105]. Another study also showed that *L. casei* supplementation reduced the levels of proinflammatory cytokines (TNF-α, IL-6 and IL-12) and elevated the level of regulatory cytokine IL-10 in RA individuals [106]. Zamani and colleagues [145] studied the effects of probiotic supplementation (*L. acidophilus*, *L. casei* and *B. bifidum*) on the clinical and metabolic status of RA patients. This supplementation improved the Disease Activity Score in 28 joints (scoring system to evaluate disease activity and treatment response in RA). Additionally, a significant decrease in the serum insulin levels was observed.

### 5.3. Diabetes Mellitus

Diabetes mellitus (DM) represents a worldwide public health issue, prevailing at approximately 450 million adults [146]. It is a chronic metabolic disease characterized by impaired insulin production/secretion or the action of insulin when an organism is unable to use insulin effectively. The vast majority of DM cases fall into two broad etiopathogenetic categories—type 1 and type 2 DM [147]. Type 1 DM (T1DM) is an autoimmune disease characterized by hyperglycemia, where elevated blood glucose levels are caused by insulin deficiency as a consequence of pancreatic β-cells loss. The aim in the management of T1DM is to promote healthy living and glycemic control. Pharmacological treatment is mainly focused on insulin therapy (insulin and insulin analogues); however, it also includes nutritional awareness to reduce the risk of cardiovascular disease and obesity and exercise to improve insulin sensitivity, lipid metabolism and blood pressure [148]. Type 2 DM (T2DM) is a multifactorial disease characterized by the dysregulation of carbohydrate, lipid and protein metabolism due to impaired insulin secretion by pancreatic β-cells, insulin resistance in skeletal muscle, liver and adipose tissue or both [149]. T2DM management includes lifestyle intervention (e.g., diet, exercise, moderate alcohol consumption and reduced sodium intake), along with pharmacological treatment, such as insulin sensitizers, insulin secretagogues, incretin-based therapies, sodium–glucose cotransporter 2 inhibitors and α-glucosidase inhibitors [150,151]. If antidiabetic treatment fails to normalize the levels of glycated hemoglobin (Hb1Ac), patients may be treated with insulin; however, high doses are often required [149].

In general, both T1DM and T2DM have a harmful impact on the bone quality, which manifests itself in a higher fracture risk [152]. Interestingly, low BMD is observed in T1DM, while BMD may not be affected in patients with T2DM. One study suggested [153] that T2DM is associated with a fracture risk, despite higher BMD levels and thicker femoral cortices in narrower bones. Another study [154] revealed that there is no relationship between T2DM and low BMD. Additionally, it was shown that even BMD was not significantly affected, and the incidence of osteoporosis was higher in T2DM patients [155]. Interestingly, Napoli and colleagues [156] demonstrated that the bone turnover markers (C-terminal telopeptide of type I collagen (CTX), osteocalcin and procollagen type 1 N-terminal propeptide (P1NP)) did not predict the incident fracture risk in T2DM patients. On the other hand, a study conducted by Starup-Linde et al. [157] showed that patients with T2DM displayed significantly lower levels of CTX and P1NP.

The pathophysiology of low BMD in T1DM can be explained by various effects such as reduced insulin signaling [114], growth hormone (GH) disorder and decreased IGF-1 [115], calcium and vitamin D [116] levels or elevated levels of proinflammatory cytokines [117].

It has been reported that the GM is altered in patients with DM (Table 1), as a relative abundance of several bacterial taxa was observed [109]. The most common alterations in patients with T1DM include bacterial species such as *Bacteroides* spp., *Streptococcus* spp., *Clostridium* spp., *Bifidobacterium* spp., *Prevotella* spp., *Staphylococcus* spp., *Blautia* spp., *Faecalibacterium* spp., *Roseburia* spp. and *Lactobacillus* spp. [107]. Pellegrini and colleagues [118] discovered an association between inflammatory markers and specific bacterial taxa. They found an increased gene expression of the cytokines CCL13, CCL19 and CCL22; chemokine receptor CCR2; cyclooxygenase 2; IL-4 receptor; CD68; pentraxin-3; TNF-α and vascular endothelial growth factor A. Their immunohistochemical analysis (performed on biopsy samples of the duodenal mucosa) also confirmed T1DM—a specific inflammatory condition. In addition, the duodenal mucosal microbiome included increased Firmicutes and a Firmicutes/Bacteroidetes ratio and a decrease in Proteobacteria and Bacteroidetes. Another study confirmed that T1DM is associated with a reduced microbiota diversity (with a significant increase in the relative abundance of *Bacteroides*, *Ruminococcus*, *Veillonella*, *Blautia* and *Streptococcus* genera and a lower relative abundance of *Bifidobacterium*, *Roseburia*, *Faecalibacterium* and *Lachnospira*). Many studies, focusing on the relationship between GM and T2DM, have provided diverse findings. Several researchers [119,120,121,122,123,124,125,126] indicated that the genera of *Bifidobacterium*, *Bacteroides*, *Faecalibacterium*, *Akkermansia* and *Roseburia* were negatively associated with T2DM, and the genera of *Ruminococcus*, *Fusobacterium* and *Blautia* were positively associated. Interestingly, the metagenome-wide association study based on the deep next-generation shotgun sequencing of stool sample DNA by Qin et al. [110] showed that patients with T2DM had only moderate bacterial dysbiosis, but there was a decline in butyrate-producing bacteria. Moreover, the findings of this study suggested a “functional dysbiosis” rather than the existence of a specific microbial species that is directly related to the pathophysiology of T2DM.

There is a growing interest in probiotic supplementation for the management and treatment of DM. Kumar et al. [111] investigated the effect of probiotic therapy (multi-strain probiotic Visbiome^®^ containing *L. paracasei, L. plantarum, L. acidophilus, L. delbrueckii, B. longum, B. infantis, B. breve and Streptococcus thermophilus*) in children with T1DM. They found a significant decrease in HbA1c (glycated hemoglobin) and decline in the total and bolus insulin dose. Other studies [112,127] examined the impacts of *Lactobacillus rhamnosus* GG and *Bifidobacterium lactis* Bb12 on β-cell function in children with newly diagnosed T1DM. However, no significant effect in maintaining the residual pancreatic β-cell function was found. The beneficial impacts of probiotic supplementation in patients with T2DM could be mediated by improving the gut integrity and peripheral insulin sensitivity, decreasing the systemic levels of LPS (lipopolysaccharide) and increasing the incretins [128]. Interestingly, *L. rhamnosus* GG improved the insulin sensitivity and reduced the lipid accumulation by stimulating adiponectin secretion in an animal model [129]. Kobyliak and colleagues [158] revealed that probiotic therapy using the multi-probiotic “Symbiter” (containing 14 probiotic bacteria genera) modestly improved the insulin resistance in individuals with T2DM.

### 5.4. Obesity

Overweight and obesity are identified as abnormal or excessive fat accumulation that can lead to impaired health. Nowadays, the prevalence of obesity is on the rise, as well as obesity-related diseases [159]. Excessive amounts of fat are associated with cardiovascular disease, T2DM, hypertension, stroke, dyslipidemia and some types of cancers and might be a risk factor for osteoporosis and fragility fractures [160,161]. Adipose tissue is not just a passive reservoir of fat, but it is now considered as an active endocrine organ involved in the modulation of energy homeostasis. It secretes numerous cytokines, such as IL-6 and TNF-α and fat-derived mediators, including resistin, leptin and adiponectin, which are also involved in bone metabolism. In addition, adipose tissue is also a source of aromatase (which catalyzes the synthesis of estrogens); hence, it is an important source of estrogen in postmenopausal women [161]. The interventions for weight control and the treatment of obesity include dietary programs; medical nutrition therapy; exercise/physical activity; behavior therapy; pharmacotherapy (e.g., orlistat, phentermine/topiramate, lorcaserin, naltrexone/bupropion and liraglutide); bariatric surgery or a combination of these treatments [133].

Pinart and colleagues [134] in their review focused on the composition of GM in obese and non-obese adults. Interestingly, in 9 out of 22 studies, there was a significantly lower alpha diversity in obese adults; however, 7 studies did not reveal a significant difference. In obese adults, significantly more *Firmicutes* and fewer *Bacteroidetes* were observed. Additionally, lower relative abundances of *Bifidobacterium* and *Eggerthella* and higher abundances of *Acidaminococcus*, *Anaerococcus*, *Catenibacterium*, *Dialister*, *Dorea*, *Escherichia-Shigella*, *Eubacterium*, *Fusobacterium*, *Megasphera*, *Prevotella*, *Roseburia*, *Streptococcus* and *Sutterella* were recorded in obese adults. In some studies, the genera *Staphylococcus* and *Clostridium*, which belong to the phylum Firmicutes, have been shown to be positively associated with obesity. The phylum Firmicutes contains many butyrate-producing species, and the increase in butyrate and acetate synthesis may contribute to an increase in energy harvest in obese subjects [130]. In addition, *Bifidobacterium* has been shown to have an inverse relationship with obesity in pregnant women and children, possibly due to the deconjugation of bile acids, which may reduce fat absorption [135,136]. Table 1 provides this information.

Manipulation of the GM can be a novel approach in preventing pathological bone loss in obese patients. Behera et al. [137] examined the effect of probiotic supplementation (previously mentioned commercially available probiotics VSL#3^®^) on mitochondrial biogenesis and bone homeostasis in obese mice fed a high-fat diet. The results showed that probiotic therapy increased the trabecular bone volume and bone mechanical strength. The treatment also prevented gut inflammation and improved osteoblast mineralization, as there was an increase of mitochondrial transcription factor A expression in osteoblasts by promoting Kdm6b/Jmjd3 histone demethylase, which inhibits H3K27me3 epigenetic methylation at the Tfam promoter. Another study reported a loss of beneficial Bifidobacteria in obese mice, while the proinflammatory species were increased [162]. Inflammation can further cause a macrophage migration to the synovium accelerating knee OA. Additionally, oligofructose (nondigestible prebiotic fiber) supplementation restored the key microbes (particularly *Bifidobacterium pseudolongum*), reduced the colonic macrophage cell signature and decreased the systemic and knee joint inflammation [162].

### 5.5. Bone Cancer

Primary bone cancers are rare, and they represent a heterogeneous group of malignancies, including osteosarcoma, chondrosarcoma and Ewing sarcoma, as the most common forms of bone cancer. The pathogenesis and origin of most bone tumors is unclear [163]. However, it has been hypothesized that osteosarcoma originate from malignant primitive mesenchymal cells that differentiate into osteoblasts, and Ewing sarcoma could be derived from undifferentiated, primitive neuroectodermal or neural crest cells. Chondrosarcoma is a cartilage-producing bone tumor, occurring mostly in the pelvis, proximal long bones, ribs, scapula and vertebrae, and its malignant cell origin are chondrocytes [164]. In addition, bone metastases are a common complication of many types of cancers occurring in patients with breast, prostate, lung, renal or thyroid cancers [165]. The treatment options for bone tumors include chemotherapy (for osteosarcoma–cisplatin, doxorubicin, methotrexate; for Ewing sarcoma–cyclophosphamide, doxorubicin and etoposide); radiation therapy and surgical management [164].

Wenhui and colleagues [166] compared the GM profiles from breast cancer patients with no metastasis (w/oBMs)*,* breast cancer patients with bone metastasis (BMs) and control individuals (Cs). They found that the community diversity was the lowest in patients with BMs. In these patients, a lack of *Megamonas* and *Akkermansia* was noted. *Streptococcus*, *Campylobacter* and *Moraxellaceae* showed higher abundances in the w/oBM and BM groups compared to the Cs. However, there are not enough studies focusing on the GM composition in bone cancer. The therapeutic value of probiotic consumption by showing an increase in cancer inhibitors and a decrease in cancer inducers has been revealed [167], but very few or no reports exist on the treatment of bone tumors with probiotics.

## 6. The Role of Gut Microbiome in Drug Response

The response to drugs can vary widely between individuals, and the role of GM in this variability is increasingly appreciated. A growing number of reports in the literature increase the level of evidence that commonly prescribed drugs that significantly affect bone homeostasis may interact with other physiological systems, such as the GM, which is essential for optimal bone function. Given the growing body of evidence linking healthy GM to bone homeostasis, the therapeutic benefits of commonly prescribed drugs and supplements, many of which are known to alter the GM [168,169]. The mechanisms of action may include drug disposition via microbial metabolism, interference by microbial metabolites or the modification of host enzymes. The current knowledge has been obtained from both animal and human studies. Unfortunately, the effect on the gut microbiome is unknown for several drugs, including bisphosphonates, approved selective estrogen receptor modulators (SERMs, e.g., raloxifene) and approved selective estrogen receptor downregulators (SERDs, e.g., fulvestrant). Selected pharmacological drugs for the treatment of bone-related diseases and also their impacts on GM are summarized in Table 2.

## 7. Conclusions

The GM plays a key role in numerous physiological functions. It can also influence bone homeostasis via several mechanisms, (e.g., immune and endocrine functions, host metabolism and the gut–brain axis). T- and B-lymphocytes can regulate bone remodeling through the expression of cytokines, as well as RANKL and OPG. SCFAs are able to reduce OC formation and resorption activity. The components of the endocrine system control a balance between bone formation and bone loss, and the maintenance of calcium and phosphate homeostasis. Specific molecules, such as brain-derived serotonin, can positively regulate bone development. 

During dysbiosis, GM is consistent with the development of various chronic diseases that can also adversely affect the bone quality and bone health. Significant changes in the GM composition in osteoporosis, osteoarthritis, rheumatoid arthritis, diabetes, obesity and bone cancer were highlighted in this review and summarized in Table 1. GM manipulation may become a future adjuvant therapy in the prevention of many chronic diseases. Therefore, the favorable impacts of probiotic supplementation in each of the above-mentioned diseases are reported in Table 1, but further studies are needed to clearly clarify its effectiveness. Recent evidence suggests that GM is responsible for the direct and indirect effects on drug efficacy. For this reason, various GM changes and interactions associated with the treatment of bone-related diseases are listed in Table 2.

## Figures and Tables

**Table 2:** Summary table of various GM alterations and interactions related to the treatment of bone-related diseases.

Disease	Selected Drugs	GM Alterations Associated with the Treatment	Interactions between the Treatment and GM	References
osteoporosis	bisphosphonates	not known	supposed GM-related immunosuppression via inhibition of the mevalonate pathway	[70]
	estrogen therapy	AS: increase in *Clostridium, Turicibacter, Romboutsia, Parabacteroides* decrease in *Kineothrix, Ruminococcus, Muribaculum*	estrogen promotes microbiota, which improves T-regulatory cell function and suppresses inflammation; GM influences estrogen metabolism	[170,171]
	raloxifene (SERMs)	not known	lasofoxifene, a SERM not approved for clinical use, affects the composition and biodiversity of GM	[172]
osteoarthritis	ibuprofen (NSAIDs)	HS: increase in families Propionibacteriaceae, Pseudomonadaceae, Puniceicoccaceae, Rikenellaceae, Acidaminococcaceae, Enterococcaceae, Erysipelotrichaceae	NSAIDs inhibit some positive effects of microbiota through enzyme inhibition.	[173]
	prednisone (glucocorticoids)	AS: increase in *Anaerobacterium* decrease in *Eisenbergiella*, *Alistipes*, *Clostridium*	microbiota-derived SCFAs might be involved in the pharmacology of prednisone	[174]
rheumatoid arthritis	methotrexate	HS: increase in *Bacteroides*, *Faecalibacterium*, *Clostridia* decrease in *Haemophilus*, *Enterobacteriales*, *Bacteroidetes*	GM includes dihydrofolate reductase enzyme, so it can modulate methotrexate metabolism; at the same time, methotrexate can modulate microbial metabolism	[175]
	dexamethasone (glucocorticoids)	AS: increase in *Lachnospiraceae*, *Oscillibacter*, *Ruminococcaceae*, *Ruminiclostridium*, *Anaerotruncus*, *Butyricicoccus, Enterobacteriaceae, Escherichia Shigella, Gammaproteobacteria, Enterobacteriales, Anaerofustis, Erysipelotrichaceae* decrease in *Lactobacillus, Enterorhabdus, Pseudomonas, Clostridium*	GM directly mediates the therapeutic efficiency and side effects of glucocorticoids	[176,177]
T1DM	insulin	not known	oral insulin administration had no effect on GM, possibly due to the insulin degradation in the intestine, and its low bioavailability	[178]
T2DM	metformin	HS: increase in *Megamonas*, *Escherichia/Shigella*, *Klebsiella*, *Blautia*, *Fusobacterium, Bifidobacterium, Intestinibacter* decrease in *Alistipes, Bacteroidetes*	metformin clinical benefits are partly mediated by bacteria-specific mechanisms such as glucose-SGLT1-sensing glucoregulatory pathway	[179,180]
obesity	orlistat	AS: increase in *Pseudomonas*, *Rhodococcus*, *Roseburia*, *Acetivibrio*	orlistat showed enrichment in genes involved in the endocrine and lipid metabolism including ALA metabolism; GM plays a role in the metabolism of ALA to conjugated linolenic acid, which was documented to have antiadipogenic effects	[181]
bone cancer	doxorubicin	AS: increase in *Faecalibaculum*, *Dubosiella*, Lachnospiraceae decrease in *Allobaculum*, *Muribaculum*, *Lachnoclostridium*	doxorubicin application is accompanied by variation of metabolism processes such as amino acid metabolism, glycan biosynthesis and metabolism, lipid metabolism, and other secondary metabolites	[182]

GM—gut microbiome; AS—animal studies; HS—human studies; T1DM—type 1 diabetes mellitus; T2DM—type 2 diabetes mellitus; SERMs—selective estrogen receptor modulators; NSAIDs—non-steroidal anti-inflammatory drugs; SCFAs—short-chain fatty acids; SGLT1—sodium/glucose cotransporter 1; ALA—alpha-linolenic acid.

## Data Availability

Not applicable.
